# Compensation of PVT Variations in ToF Imagers with In-Pixel TDC

**DOI:** 10.3390/s17051072

**Published:** 2017-05-09

**Authors:** Ion Vornicu, Ricardo Carmona-Galán, Ángel Rodríguez-Vázquez

**Affiliations:** Instituto de Microelectrónica de Sevilla, IMSE-CNM (CSIC-Universidad de Sevilla), Avda. Américo Vespucio s/n, Parque Científico y Tecnológico de La Cartuja, Seville 41092, Spain; rcarmona@imse-cnm.csic.es (R.C.-G.); angel@imse-cnm.csic.es (Á.R.-V.)

**Keywords:** PVT compensation, in-pixel time-to-digital converter (TDC), time-gating, time-of-flight (ToF), single-photon avalanche-diode (SPAD)

## Abstract

The design of a direct time-of-flight complementary metal-oxide-semiconductor (CMOS) image sensor (dToF-CIS) based on a single-photon avalanche-diode (SPAD) array with an in-pixel time-to-digital converter (TDC) must contemplate system-level aspects that affect its overall performance. This paper provides a detailed analysis of the impact of process parameters, voltage supply, and temperature (PVT) variations on the time bin of the TDC array. Moreover, the design and characterization of a global compensation loop is presented. It is based on a phase locked loop (PLL) that is integrated on-chip. The main building block of the PLL is a voltage-controlled ring-oscillator (VCRO) that is identical to the ones employed for the in-pixel TDCs. The reference voltage that drives the master VCRO is distributed to the voltage control inputs of the slave VCROs such that their multiphase outputs become invariant to PVT changes. These outputs act as time interpolators for the TDCs. Therefore the compensation scheme prevents the time bin of the TDCs from drifting over time due to the aforementioned factors. Moreover, the same scheme is used to program different time resolutions of the direct time-of-flight (ToF) imager aimed at 3D ranging or depth map imaging. Experimental results that validate the analysis are provided as well. The compensation loop proves to be remarkably effective. The spreading of the TDCs time bin is lowered from: (i) 20% down to 2.4% while the temperature ranges from 0 °C to 100 °C; (ii) 27% down to 0.27%, when the voltage supply changes within ±10% of the nominal value; (iii) 5.2 ps to 2 ps standard deviation over 30 sample chips, due to process parameters’ variation.

## 1. Introduction

Arrayable single-photon avalanche-diodes (SPADs) are available in complementary metal-oxide-semiconductor (CMOS) technologies, gaining popularity in the development of 3D image sensors [1]. Thanks to their ability to accurately detect the arrival of single photons, they can be employed to perform direct time-of-flight (ToF) measurements [2]. This is an additional feature with respect to integrating photodiodes [3], which calculate ToF by using indirect estimation methods. In order to capture a depth map of the scene, SPADs are arranged in a bi-dimensional array that can effectively associate an estimation of the ToF to each point in the image. Direct ToF requires a time-to-digital converter (TDC), able to timestamp the onset of the avalanche following the arrival of a photon, while indirect ToF [4] estimation can still be done by photon counting. Apart from 3D imaging, direct ToF measurement can be also applied to positron emission tomography (PET) [5,6] and to other biomedical techniques using a faint light source such as fluorescence lifetime imaging microscopy (FLIM) [7]. Ultimately, all these applications require statistics building of the photon arrival time which involves a lot of measurements. Therefore, high speed is a key feature of the direct ToF CMOS image sensors (dToF-CIS). Under these circumstances, perhaps the most appealing sensor architecture is the one employing per-pixel TDCs. When it comes to achieving sub-nanosecond time resolution without using several GHz clock references, time interpolation techniques must be applied. The time resolution is inversely proportional to the frequency and phase number of the clock reference. In other words, the same time resolution can be achieved with a lower frequency and higher number of phases. Therefore the time-to-digital conversion is separated in two steps: the coarse conversion made by a counter driven by the first clock phase and the fine conversion carried out by a fine interpolator. It can be employed either at the chip or pixel level. The first approach has been developed in [8,9]. A global delay-locked loop (DLL) provides 16 phases distributed to each pixel of the array. At the end of the conversion time, all phases are sampled by 16 latches based on sense amplifiers. Nevertheless, this implementation eventually implies a large number of interconnection lines, besides the TDCs output bus. This inconvenience is circumvented by routing a single phase clock to each pixel [1]. Thus, a clock reference of 280 MHz is globally generated by a DLL on-chip. It is doubled in-pixel to obtain a coarse time resolution of 1.78 ns which is further improved by a fine interpolator. In both implementations, high speed signals have to be uniformly distributed to the pixel-array. Another approach is to locally generate the high speed multiphase clock by means of per-pixel ring oscillators whose frequency is controlled by an on-chip phase-locked loop (PLL) [10]. Our design is based on a similar architecture. However the building blocks are innovative, such as: the prompt active quenching/recharge circuit with a time gated front-end; the start-stop logic of the TDC [11,12]; the voltage-controlled pseudo-differential ring-oscillator (VCRO) with a novel frequency control scheme [13]. Note that in this architecture, the uniformity of the array is given by the pixel-to-pixel mismatch.

The design of a dToF-CIS has to take into account system-level aspects that affect its overall performance. In particular when multiphase clock references are locally generated in each pixel, the impact of the process parameters, voltage supply, and temperature (PVT) variation on their stability has to be contemplated. Specifically, all these global variations end up in a deviation of the clock references’ frequency across the array. However, the frequency of the clock references is inversely proportional to the TDC time bin. In turn, the deviations of the time bins lead to a deviation of the TDCs gain, hence a deviation of the output code and estimated distance in depth map imaging or 3D ranging applications. 

The specific contribution addressed in this paper is related to the detailed mathematical analysis of PVT variations in dToF-CIS with in-pixel TDCs. The design and characterization of the control architecture able to effectively compensate the effects of PVT variations is provided as well. The validity of the oscillation frequency model and its dependence on PVT variations have been demonstrated by comparing the predictions to the simulations and/or measurements. To the best of our knowledge, this analysis has never been thoroughly approached before. 

**Figure 1:**
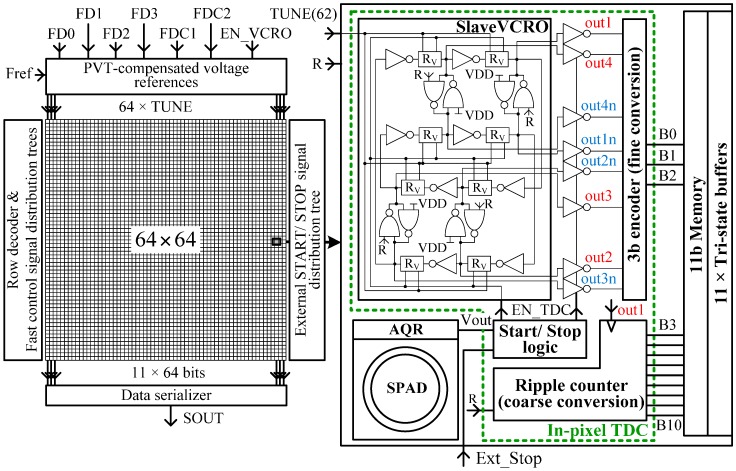
Chip diagram incorporating the compensation of process parameters, voltage supply and temperature (PVT) variations.

The dToF-CIS in question is a 64 × 64 SPAD array, with in-pixel TDCs, fabricated with standard 0.18 μm CMOS technology (Figure 1). Preliminary results of the optical and electrical characterization of the pixels’ array are reported in [11]. The TDCs employ the phase interpolation strategy based on a VCRO. Thus a ripple counter generates the coarser 8 bits of the timestamp, while the finer 3 bits are determined by properly encoding the phases of the VCRO. The resulting timestamp is stored by an in-pixel 11 bits static random-access memory (RAM) for further readout. The frequency of the VCRO array is controlled by a PLL integrated on-chip with double functionality: program the time resolution of the TDC array and compensate the multiphase clock reference against PVT variations. The imager has been integrated into a SPAD camera prototype. The system is built out of the following components: (i) the SPAD camera main board containing the housing of the SPAD imager and lens, voltage references, filters, one crystal oscillator which provides the clock reference of the PLL, and several test points; (ii) the power supply which provides 1.8 V, 3.3 V, and a variable voltage of 11–12 V for the array of SPADs; (iii) a field-programmable gate array (FPGA) board which implements the control signals, noise cancellation, and real-time 3D image reconstruction at 1000 fps; (iv) a picosecond laser. The camera is aimed for depth map imaging and photon counting applications.

The rest of the paper is organized as follows. The next section thoroughly describes the operation of the in-pixel TDC and the PLL-based global compensation scheme. The third section provides the theoretical background to understand the effect of PVT variations on the TDCs time bin. The fourth section reports several experimental results such as the time resolution programmability and the jitter of the in-pixel time interpolator. Moreover, measurements of the dependence of the TDCs time bin on the PVT variations are also shown, validating the compensation method. The models of oscillation frequency and process parameters’ variation are compared to the experimental results. The last section is dedicated to the conclusions. 

## 2. In-Pixel Time-to-Digital Converter

### 2.1. Time Bin Control Scheme

Direct ToF estimation requires accurate determination of the time interval delimited by the output pulse of the SPAD and the synchronization of the pulsed laser. For this to be carried out, a TDC is incorporated into each pixel. In order to achieve sub-nanosecond accuracy in CMOS, counting clock edges would be insufficient. The counter needs to be combined with time-interpolation techniques. In our case, each TDC (Figure 1—pixel inset: In-pixel TDC) is built by a coarse 8 bits ripple-counter, a VCRO and an encoder that generates the finest 3 bits from the phases of the VCRO, plus some control logic. A reverse start-stop scheme is employed to save power. The VCRO is started by the first positive edge of Vout, signaling a triggering of the SPAD detector. Consequently the EN_TDC signal is enabled. Later on, the VCRO is stopped by the positive edge of the Ext_Stop synchronization signal, causing EN_TDC to be disabled. At this time, the ripple counter and the 8 phases of the oscillator are frozen. The most significant 8 bits of the ToF estimation are contained by the counter, while the VCRO’s phases are encoded to provide the least significant 3 bits. The conversion result is available at the very end of the measured time interval which makes this scheme appropriate for applications which require a high conversion rate.

**Figure 2:**
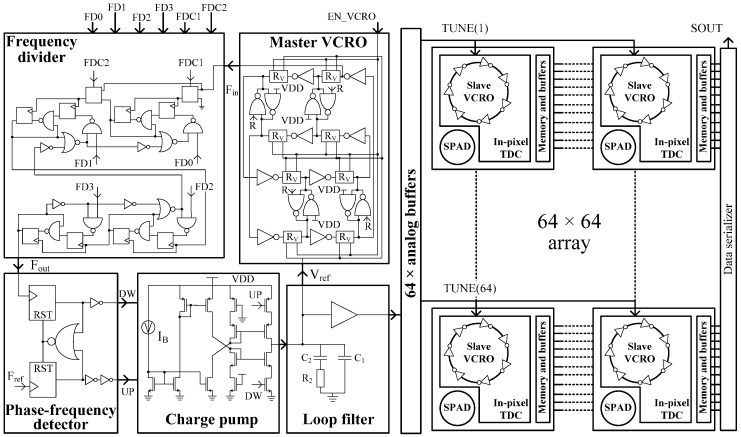
Compensation block diagram of global PVT variations.

The array of VCROs is driven by the voltage labeled as TUNE. It is provided by a global compensation scheme implemented by an on-chip PLL (Figure 2). A master VCRO is locked by the PLL. Whenever a PVT variation occurs, the loop acts to correct it by adjusting the voltage on the loop filter in order to keep the same oscillation frequency. Consequently, the oscillation frequency of the slave VCROs follows the master VCRO (Figure 2—inset: Master VCRO). It is worth mentioning that this compensation technique works if the gradient of the PVT variations is uniformly distributed across the array. Moreover, the ripple of the TUNE voltage causes a deviation of the TDC time bin. This implication can be written as:

(1) $$∆V_{ref}=\frac{\sqrt{1+{\left(8K_{VCRO}∆T_{bin}V_{ref}\right)}^{2}}-1}{4K_{VCRO}∆T_{bin}}$$

where $∆V_{ref}$ is the ripple of the loop filter output, $∆T_{bin}$ is the deviation in the TDC time bin, $K_{VCRO}$ is the VCRO gain, and $V_{ref}$ is the average output of the loop filter.

### 2.2. Pseudo-Differential VCRO

**Figure 3:**
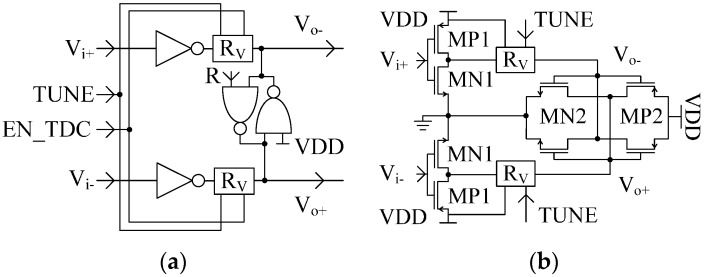
Delay cell (**a**) Block diagram (**b**) Equivalent schematic when signals R and EN_TDC are connected to the voltage supply VDD.

The ring oscillator is the core building block of the TDC. It gives the clock for the ripple-counter generating the most significant bits of the conversion, while providing the phases for the encoder to obtain the least significant bits of the conversion by phase interpolation. The proposed scheme is a 4-stage pseudo-differential VCRO, also suitable to be implemented in FPGA [14]. The block diagram of the VCRO is depicted in Figure 1—pixel inset: Slave VCRO. Notice that the reset signal forces the oscillator to start each time with the same phase. This auto-alignment minimizes the start-up error which affects the time accuracy of the TDC. The selection of the number of stages is a matter of trade-off between the number of interpolation phases and the area. Thus, considering an oscillation frequency of 850 MHz, four stages are enough to obtain a time resolution below 150 ps. The novelty of this VCRO scheme relies on the highly linear frequency control scheme, which is based on the variable resistance of a transmission gate, represented by the block “R_V_” in Figure 3. 

**Figure 4:**
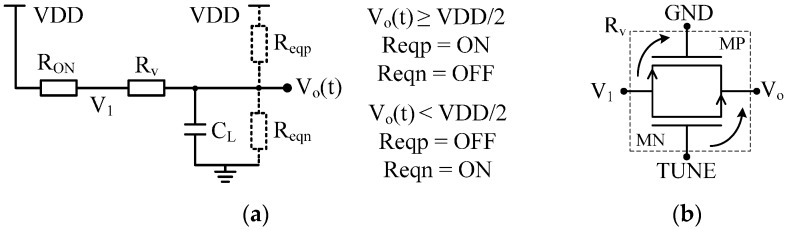
(**a**) Half circuit of the delay cell when the output capacitance $C_{L}$ is charging; (**b**) schematic of the R_V_ block when EN_TDC is connected to VDD.

Let us consider the schematic of the delay cell depicted in Figure 3. The reset signal, R, is disabled, being set to VDD. The half circuit of the delay cell (Figure 4a) can be employed to approximate the oscillation frequency of the VCRO. By solving the differential equation of the step response, the output voltage is:

(2) $$\{\begin{array}{c} V_{o}\left(t\right)=\frac{R_{eqn}}{R_{V}+R_{ON}+R_{eqn}}\mathrm{VDD}\left(1-e^{-\frac{t}{\tau_{n}}}\right),V_{o}\left(t\right)<\frac{VDD}{2}\\ V_{o}\left(t\right)≅\mathrm{VDD}\left(1-\frac{R_{eqn}}{R_{eqn}-R_{V}-R_{ON}}e^{-\frac{t}{\tau_{p}}}\right),V_{o}\left(t\right)\geq \frac{VDD}{2} \end{array}$$

where the time constant $\tau_{n}$ and $\tau_{p}$ have the following forms:

(3) $$\{\begin{array}{c} \tau_{n}=\frac{(R_{V}+R_{ON})R_{eqn}}{R_{V}+R_{ON}+R_{eqn}}C_{L}\\ \tau_{p}=\frac{(R_{V}+R_{ON})R_{eqp}}{R_{V}+R_{ON}+R_{eqp}}C_{L} \end{array}$$

$R_{eqn}$, $R_{eqp}$, $R_{ON}$, and $R_{V}$ are the equivalent resistances (including the cross coupled inverters) loading the output node of the delay cell, the resistance of MP1 (Figure 3b) working as a switch, and the equivalent variable resistance of the transmission gate labeled “R_V_” (see Figure 4), respectively. 

The large signal oscillation frequency can be written as [15]:

(4) $$f_{o}=\frac{1}{2Mt_{d}}$$

where $t_{d}$ is the propagation delay and M is the number of delay cells. It is defined as the time lapse between the ideal input step and the moment when the output ramp crosses the trip point of the next delay cell. Considering that the switching point of the delay cell is at VDD/2 and applying Equation (2), $t_{d}$ is written as:

(5) $$t_{d}=\tau_{n}\ln\frac{2R_{eqn}}{R_{eqn}-R_{V}-R_{ON}},\;R_{eqn}>R_{V}+R_{ON}$$

Note that $R_{eqn}$ and $R_{eqp}$ depend on the strength of the positive reaction of the cross-coupled inverters. 

## 3. Effect of PVT Variations on the Time Bin

### 3.1. Temperature Dependence

Equations (4) and (5) are employed to analyze the dependence of $f_{o}$ on temperature variations. The carrier mobility and threshold voltage of the transistors along the signal path depend on the temperature (Equation (6)) which involves changes of $t_{d}$. From now on, let us consider that the threshold voltages of both NMOS (N channel metal-oxide semiconductor) and PMOS (P channel metal-oxide semiconductor) transistors are positive, i.e., $V_{T_{N}}>0$ and $V_{T_{P}}>0$.

(6) $$V_{T_{N,P}}\left(T\right)=V_{T0_{N,P}}\left(T_{0}\right)\left[1+TCV_{T_{N,P}}\left(T-T_{0}\right)\right],\;\mu_{n,p}\left(T\right)=\mu_{0n,p}\left(T_{0}\right){\left(\frac{T}{T_{0}}\right)}^{-1.5}$$

where the temperature coefficient for both transistors is negative [16]:

(7) $$TCV_{T_{N,P}}=\frac{1}{V_{T_{N,P}}}\cdot \frac{\partial V_{T_{N,P}}}{\partial T}<0$$

Then the temperature derivative of $f_{o}$ is:

(8) $$\frac{\partial f_{o}}{\partial T}=-\frac{1}{2Mt_{d}^{2}}\frac{\partial t_{d}}{\partial T},\;t_{d}≅\tau_{n}\left(\ln2+\frac{R_{V}+R_{ON}}{R_{eqn}}\right),\;\mathrm{if}\;R_{eqn}\gg R_{V}+R_{ON}$$

which involves the computation of the temperature derivative of $t_{d}$:

(9) $$\frac{\partial t_{d}}{\partial T}=\frac{\partial \tau_{n}}{\partial T}\left(ln2+\frac{R_{V}+R_{ON}}{R_{eqn}}\right)+\tau_{n}\frac{\partial }{\partial T}\left(\frac{R_{V}+R_{ON}}{R_{eqn}}\right)$$

Rewriting Equation (9), we find that the sign of $\partial t_{d}/\partial T$ depends on the sign of $\partial R_{eqn}/\partial T$, $\partial R_{V}/\partial T$, and $\partial R_{ON}/\partial T$:

(10) $$\frac{\partial t_{d}}{\partial T}≅\frac{C_{L}}{R_{V}+R_{ON}+R_{eqp}}\left[\frac{{\left(R_{V}+R_{ON}\right)}^{2}\frac{\partial R_{eqn}}{\partial T}+R_{eqn}^{2}\left(\frac{\partial R_{V}}{\partial T}+\frac{\partial R_{ON}}{\partial T}\right)}{R_{V}+R_{ON}+R_{eqp}}\left(ln2+\frac{R_{V}+R_{ON}}{R_{eqn}}\right)+\left(R_{V}+R_{ON}\right)\left(\frac{\partial R_{V}}{\partial T}+\frac{\partial R_{ON}}{\partial T}\right)\right]$$

Let us suppose that $R_{eqn}$, $R_{ON}$, and $R_{V}$ are linear and the equivalent resistances are approximated as:

(11) $$R_{eqn}=\frac{1}{\beta_{MN2}\left(VDD-V_{T_{N}}\right)},\;R_{ON}=\frac{1}{\beta_{MP1}\left(VDD-V_{T_{P}}\right)}\;\mathrm{with}\;V_{SD_{MP1}}\ll 2\left(V_{SG_{MP1}}-V_{T_{P}}\right)$$

(12) $$R_{V}=R_{V,MN}||R_{V,MP},\;R_{V,MN}=\frac{1}{\beta_{MN}\left(TUNE-V_{o}-V_{T_{N}}\right)},R_{V,MP}=\frac{1}{\beta_{MP}\left(\mathrm{VDD}-V_{sat,MP1}-V_{T_{P}}\right)}\;\mathrm{for}\;V_{o}<TUNE-V_{T_{N}}$$

This assumption is made only to simplify the computation of the derivatives which are written as follows:

(13) $$\frac{1}{R_{eqn}}\frac{\partial R_{eqn}}{\partial T}=\frac{1}{VDD-V_{T_{N}}}\frac{\partial V_{T_{N}}}{\partial T}-\frac{1}{\mu_{n}}\cdot \frac{\partial \mu_{n}}{\partial T},\mathrm{and}\;\frac{1}{R_{ON}}\frac{\partial R_{ON}}{\partial T}=\frac{1}{VDD-V_{T_{P}}}\frac{\partial V_{T_{P}}}{\partial T}-\frac{1}{\mu_{p}}\cdot \frac{\partial \mu_{p}}{\partial T}$$

(14) $$\frac{\partial R_{V}}{\partial T}=\frac{1}{{\left(R_{V,MN}+R_{V,MP}\right)}^{2}}\left(R_{V,\mathrm{MP}}^{2}\frac{\partial R_{V,\mathrm{MN}}}{\partial T}+R_{V,\mathrm{MN}}^{2}\frac{\partial R_{V,\mathrm{MP}}}{\partial T}\right)$$

$$\frac{1}{R_{V,MN}}\frac{\partial R_{V,MN}}{\partial T}=\frac{1}{TUNE-V_{o}-V_{T_{N}}}\frac{\partial V_{T_{N}}}{\partial T}-\frac{1}{\mu_{n}}\cdot \frac{\partial \mu_{n}}{\partial T},\;V_{o}<TUNE-V_{T_{N}}$$

$$\frac{1}{R_{V,MP}}\frac{\partial R_{V,\mathrm{MP}}}{\partial T}=\frac{1}{\mathrm{VDD}-V_{sat,MP1}-V_{T_{P}}}\cdot \frac{\partial V_{T_{P}}}{\partial T}-\frac{1}{\mu_{p}}\cdot \frac{\partial \mu_{p}}{\partial T}$$

where $R_{V,MN}$ and $R_{V,MP}$ are the two components of the transmission gate. We have evaluated Equations (13) and (14) to find the sign of the derivatives, which is not obvious in this case. Based on simulation results, ∆T, $∆\mu_{n}$, $∆\mu_{p}$, $∆V_{T_{N}}$, and $∆V_{T_{P}}$ are of 100, −0.013, −0.005, −85 mV, and −95 mV, respectively. Under these circumstances, the derivatives from Equations (13) and (14) are positive, leading to a positive derivative of $t_{d}$. 

Hence the derivative of the oscillation frequency, $f_{o}$, and the TDC time bin, $T_{bin}$, with respect to temperature can be expressed as:

(15) $$\frac{\partial f_{o}}{\partial T}=-\frac{1}{2Mt_{d}^{2}}\frac{\partial t_{d}}{\partial T}<0$$

(16) $$T_{bin}=t_{d},\;\frac{\partial T_{bin}}{\partial T}=\frac{\partial t_{d}}{\partial T}>0$$

Equation (15) shows that $f_{o}$ decreases as the temperature rises. It will also be proved by the experimental results, in the next section. Moreover, the model of the oscillation frequency (see Equations (4) and (5)) is demonstrated in Section 4.1 by comparing it with the measurement results. 

### 3.2. Voltage Supply Dependence

In order to analyze this dependence, we use the same model for the oscillation frequency and time bin as in the previous subsection. 

Similar with the result obtained for the temperature variation, it has been found that the sign of $\partial t_{d}/\partial VDD$ depends on the sign of $\partial R_{eqn}/\partial VDD$, $\partial R_{V}/\partial VDD$, and $\partial R_{ON}/\partial VDD$:

(17) $$\frac{\partial t_{d}}{\partial VDD}≅\frac{C_{L}}{R_{V}+R_{ON}+R_{eqp}}\left[\frac{{\left(R_{V}+R_{ON}\right)}^{2}\frac{\partial R_{eqn}}{\partial VDD}+R_{eqn}^{2}\left(\frac{\partial R_{V}}{\partial VDD}+\frac{\partial R_{ON}}{\partial VDD}\right)}{R_{V}+R_{ON}+R_{eqp}}\left(ln2+\frac{R_{V}+R_{ON}}{R_{eqn}}\right)+\left(R_{V}+R_{ON}\right)\left(\frac{\partial R_{V}}{\partial VDD}+\frac{\partial R_{ON}}{\partial VDD}\right)\right]$$

Hence the derivatives of $R_{eqn}$, $R_{ON}$, and $R_{V}$ are written as follows:

(18) $$\frac{\partial R_{eqn}}{\partial VDD}=-R_{eqn}^{2}\beta_{MN2}<0,\;\mathrm{and}\frac{\partial R_{ON}}{\partial VDD}=-R_{ON}^{2}\beta_{MP1}<0$$

(19) $$\frac{\partial R_{V}}{\partial VDD}≅\frac{R_{V,\mathrm{MN}}^{2}}{{\left(R_{V,MN}+R_{V,MP}\right)}^{2}}\frac{\partial R_{V,\mathrm{MP}}}{\partial VDD}\;\mathrm{with}\;\frac{\partial R_{V,\mathrm{MP}}}{\partial VDD}=-R_{V,MP}^{2}\beta_{MP}<0$$

This time, according to Equations (18) and (19), it is obvious that the derivative of the time delay to the voltage supply is negative (Equation (17)). 

Again, evaluating the effect on the time bin by calculating the partial derivative of $T_{bin}$ to the voltage supply, we found that:

(20) $$\frac{\partial T_{bin}}{\partial VDD}=\frac{\partial t_{d}}{\partial VDD}<0$$

The time bin decreases when VDD increases. This fulfills our expectations because the oscillation frequency grows with the voltage supply. 

### 3.3. Process Parameter Variation Effect

The variation of the process parameters leads to deviations of the VCRO oscillation frequency, $∆f_{o}$. Taking into account that the VCROs are used as time interpolators for the TDCs, $∆f_{o}$ involves a deviation of the TDCs time bin, $∆T_{bin}$ (see Equation (1)). The global compensation loop is also employed to minimize $∆f_{o}$ from chip-to-chip.

(21) $$\frac{∆T_{bin}}{T_{bin}}=\frac{∆f_{o}}{f_{o}},\;T_{bin}=\frac{1}{2Mf_{o}}$$

where M is the number of pseudo-differential delay cells. Let us consider that $∆f_{o}$ is due to the deviations of the variable resistance and lumped capacitance, namely $∆R_{V}$ and $∆C_{L}$, respectively. These deviations are caused by the variation of process parameters, such as electron and hole mobility, gate oxide thickness, threshold voltage, and transistor length and width. Assume that $∆R_{V}$ and $∆C_{L}$ are normally distributed with zero mean and non-zero standard deviation. 

The chip-to-chip relative deviation of the oscillator frequency of an individual VCRO, $∆f_{o}/f_{o}$, is computed as:

(22) $${\left(\frac{∆f_{o}}{f_{o}}\right)}^{2}={\left(\frac{∆R_{V}}{R_{V}}\right)}^{2}+{\left(\frac{∆C_{L}}{C_{L}}\right)}^{2}$$

where $∆R_{V}\ll R_{V}$, $∆C_{L}\ll C_{L}$.

The contribution of ${\left(∆R_{V}/R_{V}\right)}^{2}$ can be expressed as a function of ${\left(∆V_{T}\right)}^{2}$ and ${\left(∆\beta /\beta \right)}^{2}$ that are inversely proportional to the device area [17]:

(23) $${\left(\frac{∆R_{V}}{R_{V}}\right)}^{2}=R_{V}^{2}\beta_{MN}^{2}\beta_{MP}^{2}\left\{{\left[\frac{\mathrm{TUNE}-V_{o}-V_{T_{N}}}{\beta_{MP}}\right]}^{2}{\left(\frac{∆\beta_{MN}}{\beta_{MN}}\right)}^{2}+{\left[\frac{VDD-V_{sat,MP1}-V_{T_{P}}}{\beta_{MN}}\right]}^{2}{\left(\frac{∆\beta_{MP}}{\beta_{MP}}\right)}^{2}+\frac{1}{\beta_{MP}^{2}}{\left(∆V_{T_{N}}\right)}^{2}+\frac{1}{\beta_{MN}^{2}}{\left(∆V_{T_{P}}\right)}^{2}\right\}$$

The contribution of ${\left(∆C_{L}/C_{L}\right)}^{2}$ is evaluated taking into account that $C_{L}$ is a lumped capacitor that is a sum of $\alpha_{i}C_{ox}W_{i}L_{i}$ capacitances, where $\alpha_{i}$ is either 1, 2/3, or 1/2 depending on the transistor’s operation point. The subscription i is either MN, MN1, MN2, MP, MP1, or MP2. Therefore the relative deviation of the output capacitance is written as:

(24) $${\left(\frac{∆C_{i}}{C_{i}}\right)}^{2}={\left(\frac{∆W_{i}}{W_{i}}\right)}^{2}+{\left(\frac{∆L_{i}}{L_{i}}\right)}^{2}+{\left(\frac{∆t_{ox}}{t_{ox}}\right)}^{2}$$

According to Equations (22)–(24), the larger the area of the devices, the smaller the relative deviation of $f_{o}$. The area of the transistors cannot be increased too much because the occupied area is one of the top priorities of the design. Under these circumstances, the compensation loop is successfully used to reduce the drift of $f_{o}$ due to the chip-to-chip process parameters’ variation. Further improvement can be done by applying an off-chip compensation based on look-up tables.

The proposed model is compared to the simulation and measurement results of over 30 samples. This discussion will be developed in Section 4.4.

## 4. Experimental Results

The analysis presented in the previous section is experimentally confirmed by measurements on a prototype chip. First, we will illustrate the dependence of the $T_{bin}$ on the control voltage, TUNE. Afterwards, we will display measurements on the effect of each PVT variation on $T_{bin}$, with (TUNE is an internal voltage reference provided by the PLL loop filter) and without (TUNE is an external voltage reference) activating the compensation loop.

### 4.1. Time Bin of the TDC Array

**Figure 5:**
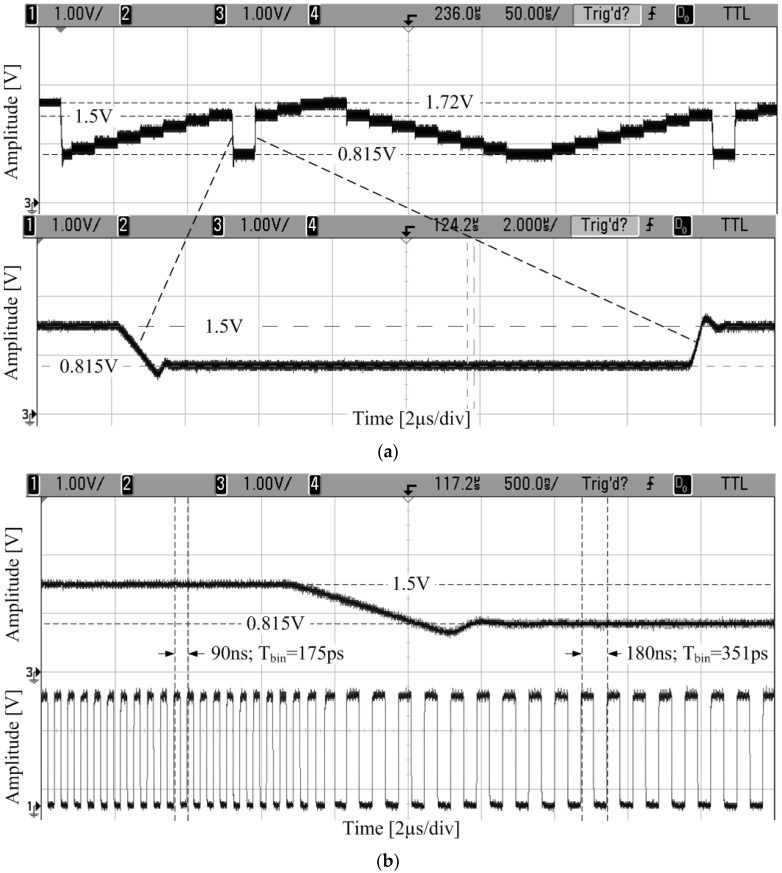
Time resolution programmability: (**a**) Reference voltage; (**b**) In-pixel voltage-controlled ring-oscillator (VCRO) frequency control.

As mentioned before, the reference voltage for the array of VCROs is provided by an on-chip PLL. Also, by changing the loop division factor, the time resolution of the sensor can be set to a different value. In our prototype chip, the time resolution of the TDCs ranges between 145 ps and 357 ps (Figure 5). This measurement has been reported as a preliminary result in [11]. The output frequency of the oscillator varies between 850 MHz and 350 MHz. The frequency divider of the PLL takes about 15.5 μs to change its configuration; then the locking time is less than 3 μs. Figure 5b shows the change in frequency of the VCRO, at pixel (64, 64) when the loop division factor changes from 15 down to 8. Therefore the oscillation frequency jumps from 711 MHz to 355 MHz. 

**Figure 6:**
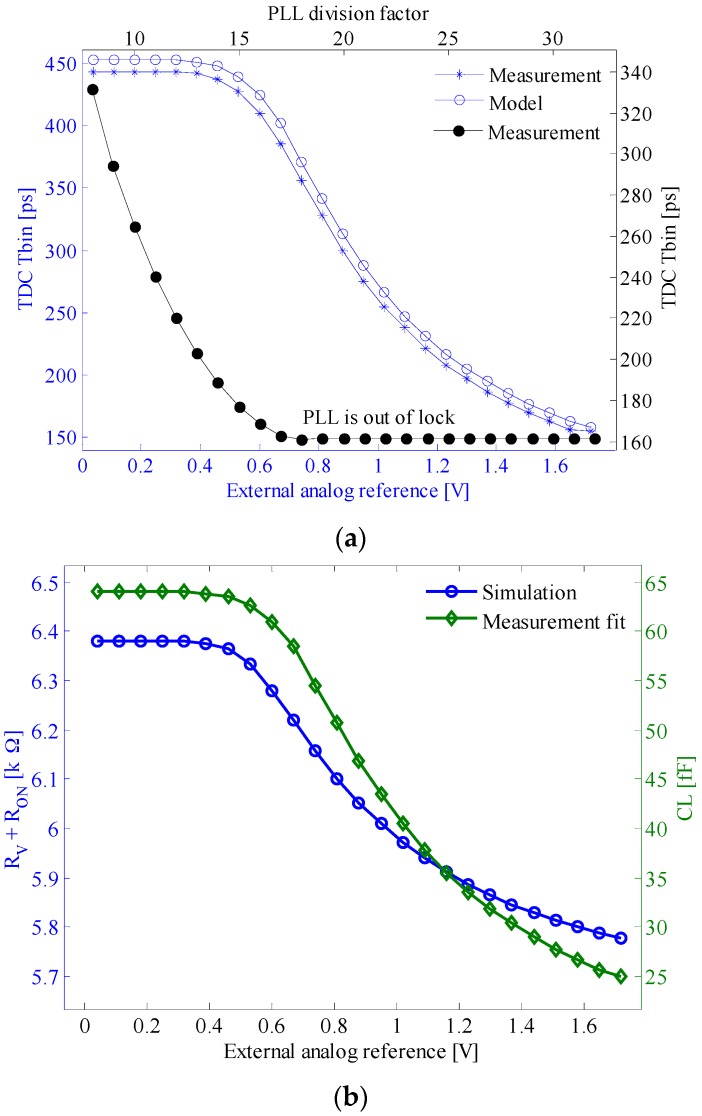
(**a**) TDC T_bin_ dependence on the frequency control voltage which is either internal (dot marker; upper-right XY axes) or external (asterisk marker for measurement and circle marker for model; lower-left XY axes). (**b**) The equivalent resistance $R_{V}+R_{ON}$ (circle marker; left Y-axis) and output capacitance $C_{L}$ (diamond marker; right Y-axis).

We have measured the TDC time bin as a function of the VCRO reference voltage, TUNE. The oscillation frequency has been analytically computed according to Equations (4) and (5). We have determined the piece-wise linearized resistances $R_{V}+R_{ON}$ (Figure 6b—circle marker) and $R_{eqn}$ from simulations. Note that $R_{ON}$ is smaller than $R_{V}$ and does not depend on the TUNE voltage; besides, $R_{eqn}$ is much larger than $R_{V}+R_{ON}$, and for simplicity we have considered a constant value of 30 kΩ. With these values, we have fitted the predicted $t_{d}$ by using Equation (5) (Figure 6a—circle marker) on the measured $t_{d}$ (Figure 6a—asterisk marker) and found the output capacitance of the delay cell, $C_{L}$ (Figure 6b—diamond marker). 

The TDC time bin as a function of PLL division factor has been measured as well (Figure 6a—dot marker). Note that the X-axis is the division factor N of the PLL instead of a voltage signal. This is because N is the actual control input which in turn is proportional to the internal reference voltage which controls the TDC array in this case. 

A first approximation of the in-pixel TDC time bin, considering that the core oscillator has an external voltage reference is:

(25) $$T_{bin}=\frac{1}{2MK_{VCRO}V_{ref}}\;=\;\frac{1}{2Mf_{o}}$$

where $K_{VCRO}$ is the core oscillator sensitivity to voltage control, $V_{ref}$, and $f_{o}$ is the nominal oscillation frequency of the VCRO. M is the number of delay cells. If the voltage reference is provided by the on-chip PLL, then the time bin is written as:

(26) $$T_{bin}=\frac{1}{2MNf_{ref,PLL}}$$

where N is the frequency division factor, and $f_{ref,PLL}$ is the PLL reference frequency. Notice that the best time bin is limited by the PLL which goes out of lock (see Figure 6a—black curve with dot marker).

**Figure 7:**
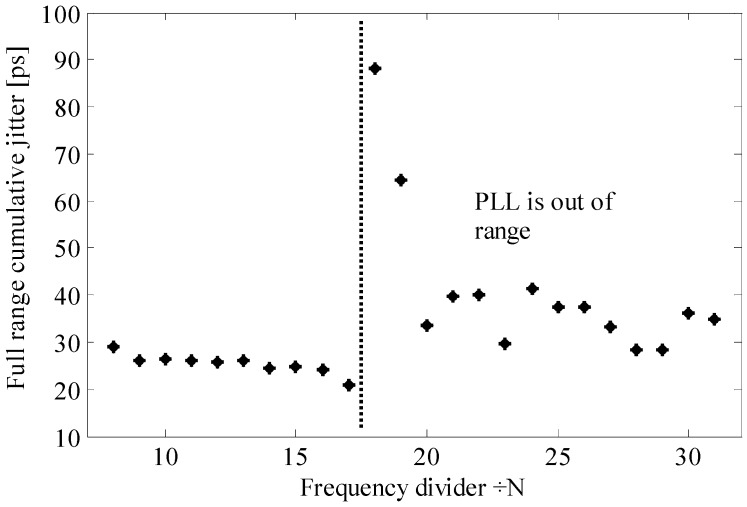
Cumulative jitter of the in-pixel VCRO, measured for a time window equal to the TDC full range; the frequency control voltage of the VCRO is connected to the internal reference provided by the phase-locked loop (PLL) loop filter.

The single shot precision of the TDC is affected by the cumulative jitter, $\sigma_{c}$, of the VCRO. In order to measure it, we selected the VCRO of a test pixel and connected its frequency control voltage to the internal reference provided by the PLL loop filter. The jitter is measured over a time window corresponding to the TDC full range, namely $2^{11}T_{bin}$. The standard deviation of $\sigma_{c}$ is depicted in Figure 7. It is worth mentioning that for division factors larger than 17, the PLL goes out of lock. Thus, the ripple on the loop filter increases. According to Equation (1), this ripple modulates the output frequency of the VCRO, and hence the $T_{bin}$. This is the reason why the jitter increases when the PLL goes out of range.

### 4.2. Compensation of Temperature Variations

**Figure 8:**
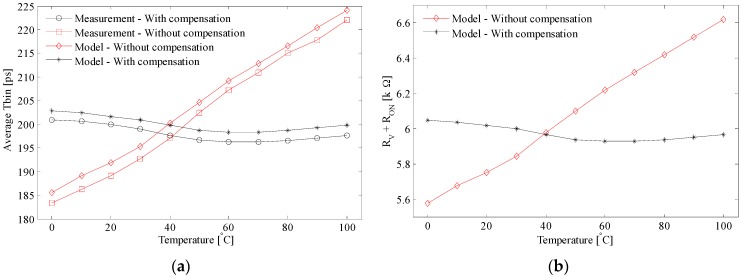
(**a**) Average $T_{\mathrm{bin}}$ dependence on temperature variation: with compensation (model—asterisk marker; measurement—circle marker) and without compensation (model—diamond marker; measurement—square marker) (**b**) Equivalent resistance $R_{V}+R_{ON}$ as a function of temperature: with compensation—asterisk marker; without compensation—diamond marker.

The time bins of the array of TDCs are sensitive to temperature variations. According to Equation (16), the TDC time bin increases when the temperature rises which it is confirmed by the measurement results (Figure 8a—square marker). Considering that $C_{L}$ is the lumped parasitic capacitor connected to the output node of the delay cell, it mainly depends on the transistors’ geometry and their operating points. Thus it is assumed to be invariant to temperature. Therefore by fitting the modeled $t_{d}$ (Figure 8a: without compensation–diamond marker; with compensation—asterisk marker) on the measured $t_{d}$ (Figure 8a: without compensation–square marker; with compensation–circle marker) we have found the dependence of the resistance $R_{V}+R_{ON}$ on temperature (Figure 8b: without compensation–diamond marker; with compensation—asterisk marker). For simplicity, $R_{eqn}$ has been considered constant. 

**Figure 9:**
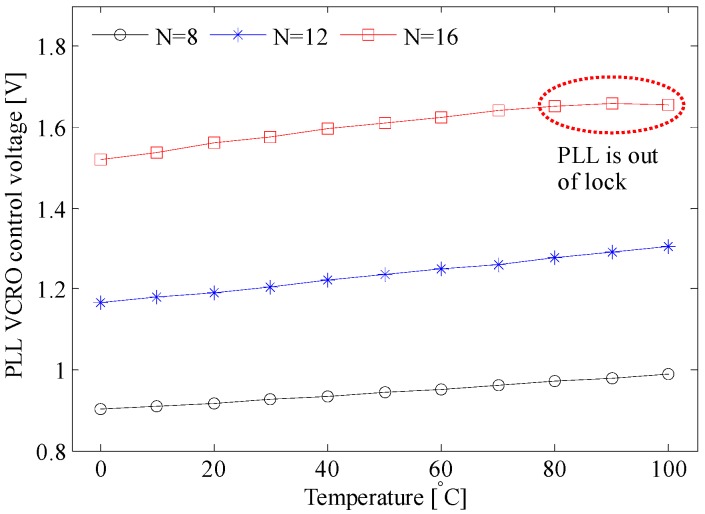
The dependence of the VCRO control voltage on the temperature variation.

**Figure 10:**
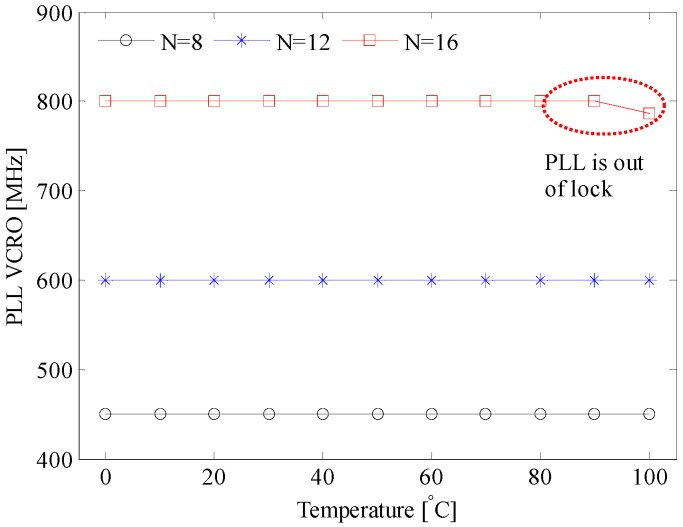
PLL behavior across the temperature variation; N is the division factor of the frequency divider.

The compensation mechanism is explained in Figure 2. As long as the PLL is locked, the loop acts as follows: if the temperature rises, the oscillation frequency $f_{o}$ of the master VCRO decreases (see Equation (15)). Consequently, the frequency control voltage increases, causing $f_{o}$ to increase. This fact is demonstrated by the measurements displayed in Figure 9 for the three different frequency division factors. Figure 10 proves that the oscillation frequency of the master VCRO is invariant to temperature. This temperature-invariant oscillation frequency is employed to generate a control voltage that feeds the frequency control input of the slave VCROs incorporated in the TDC array. The effectiveness of this compensation scheme is evaluated in Figure 8. The spreading of the $T_{bin}$ caused by temperature variation is significantly lowered from 20% down to 2.4%, with average values of 202.2 ps and 198 ps. 

In order to have a better understanding of these deviations from the application point of view, one has to convert them into time intervals and distances. Without compensation, the peak-to-peak deviation of $T_{bin}$ is of 38.7 ps which leads to an equivalent depth error of 11.88 m at a maximum distance range of 62 m. With compensation, the error decreases to 1.4 m at a maximum distance range of 60.8 m.

### 4.3. Compensation of Voltage Supply Variations

**Figure 11:**
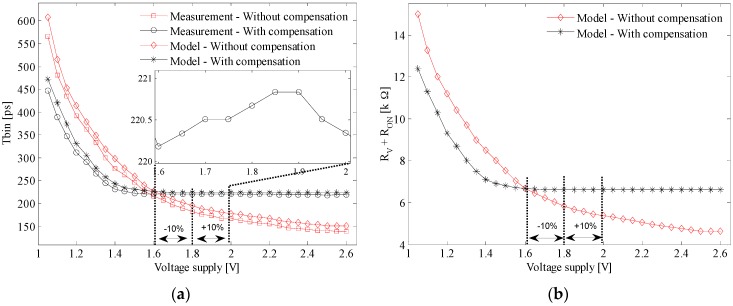
(**a**) Average $T_{\mathrm{bin}}$ dependence on VDD: with compensation (model—asterisk marker; measurement—circle marker) and without compensation (model—diamond marker; measurement—square marker) (**b**) Equivalent resistance $R_{V}+R_{ON}$ as a function of temperature: with compensated—asterisk marker; without compensation—diamond marker.

According to Equation (20), the TDC time bin or the time delay decreases when the voltage supply rises. This prediction has been confirmed by the measurement depicted in Figure 11a—square marker. Similar to temperature variation, let us consider that the $C_{L}$ and $R_{eqn}$ deviations due to VDD changes are neglected. By fitting the modeled $t_{d}$ (Figure 11a: without compensation—diamond marker; with compensation—asterisk marker) to the measured $t_{d}$ (Figure 11a: without compensation—square marker; with compensation—circle marker), we have found the dependence of $R_{V}+R_{ON}$ on the VDD variations (Figure 11b: with compensation—asterisk marker; without compensation—diamond marker). The average TDC time bin measured for the uncompensated and compensated TDC array is of 181.5 ps and 220.7 ps, respectively. Let us consider a common variation of the voltage supply of ±10% from the nominal value of 1.8 V. The spreading of the TDC time bin is remarkably reduced from 27% down to 0.27% of its corresponding nominal values. From the application point of view, this deviation translated into a depth estimation error of 15 m at a maximum distance range of 55.7 m. This error is lowered by the compensation loop down to 18.4 cm at a maximum distance range of 67.8 m.

**Figure 12:**
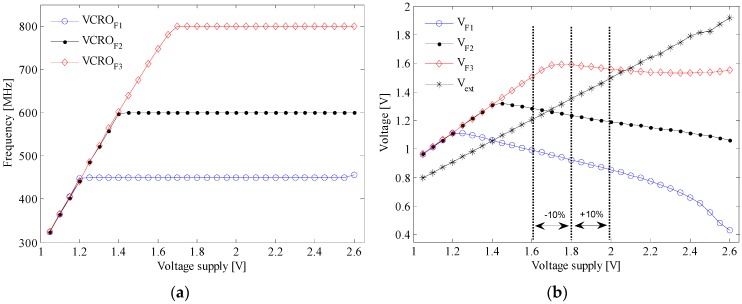
Measured synthesized frequencies’ dependence on the variation of VDD: (**a**) master VCRO output frequency and (**b**) loop filter voltage.

Note that the compensation loop stays locked for a wider range of VDD, i.e., from 1.4 V to 2.6 V. The behavior of the PLL with the variation of the voltage supply is revealed in Figure 12 as follows. Figure 12a shows that different synthesized frequencies are locked while the voltage supply varies from 1.7 V to 2.55 V. VCRO_F1, 2, 3_ are the output voltages of the control loop when N is 9, 12, and 16. These values correspond to output frequencies of 450 MHz, 600 MHz, and 800 MHz, respectively.

The graph in Figure 12b shows how the compensation loop works. V_F1, 2, 3_ are the voltages on the loop filter when N is 9, 12, and 16, respectively, and V_ext_ is an external analog voltage reference. As it has been demonstrated, the VCRO output frequency increases when the voltage supply rises. 

The loop acts to cancel this variation by decreasing the internal reference voltage taken from the loop filter. In this way, the output frequencies of the master VCRO and the array of slave VCROs are decoupled from the voltage supply variation.

### 4.4. Attenuation of the Effect of the Process Parameters’ Variation

The relative deviation of $f_{o}$ due to process variation has been modeled. It depends on the relative deviations of $R_{V}$ and $C_{L}$ which subsequently are modeled by Equations (23) and (24). The prediction of Equation (22) is compared with the simulation results and measurements (see Table 1).

**Figure 13:**
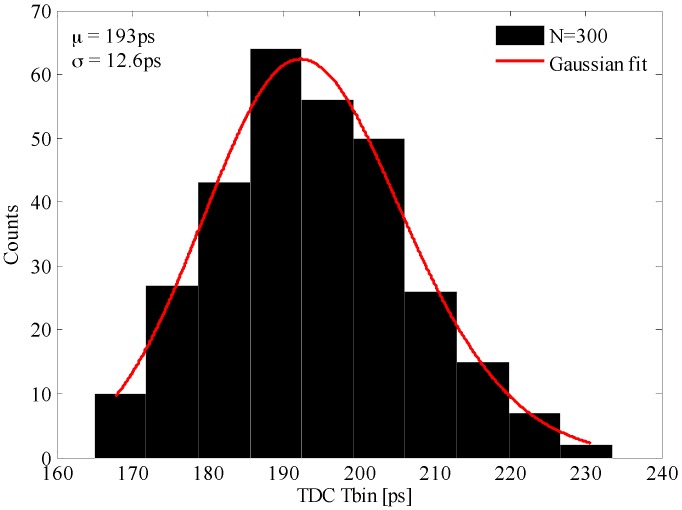
Simulated TDC $T_{\mathrm{bin}}$ deviation due to the process parameters’ variation.

This analysis is backed up by the following discussion around the measured, predicted, and experimental results. To start, a Monte Carlo post-layout simulation has been performed to determine the standard deviation of $f_{o}$ due to process variation. The simulation results are depicted in Figure 13. The mean value and standard deviation of $T_{bin}$ are 193 ps and 12.6 ps, respectively. Consequently, the standard deviation of $f_{o}$ is 42.28 MHz for an average value of 647.4 MHz. This has been computed by Equation (21). 

Hence, the simulated results have been compared to the prediction of the proposed model by Equations (22–24). The model parameters have the following values: TUNE=1.31 V, VDD=1.8 V, $V_{o}=0.9\;V$, $e_{ox}=\;35.13\;\mathrm{pF}/m$. The TUNE signal has been set such that the time bin of the TDC array falls in the middle of its range. The rest of the parameters are displayed in Table 2. They have been extracted from the previous Monte Carlo simulation.

**Figure 14:**
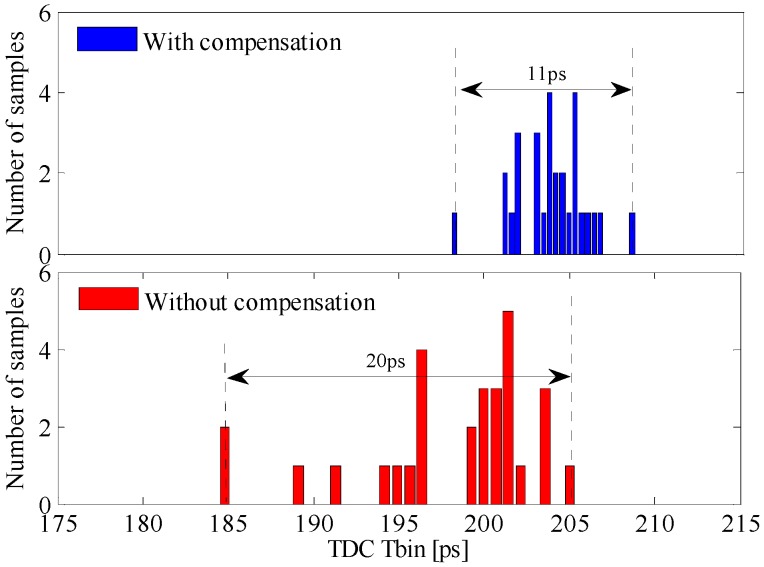
Measured process variation effect on the TDC $T_{\mathrm{bin}}$.

Ultimately, the predicted and simulated results have to be compared to the experimental results. For this purpose, 30 samples have been measured by evaluating the performance of a certain pixel. The effectiveness of the parameter variation compensation loop is proved as well. Each sample has been evaluated in two different scenarios. In the first experimental setup, we have connected the analog reference of the array of slave VCROs to an external voltage set at 1.31 V, as it was in the simulation. The second measurement setup consists of setting the analog reference to be the internal PLL loop filter voltage. The frequency divider of the PLL is set such that the output voltage given by the loop filter has to have a value close to the one chosen for the external reference voltage. The TDC time bin has been measured in both scenarios for all the samples (Figure 14). Without compensation, the time bin has a standard deviation of 5.2 ps, for an average value of 198.2 ps. With compensation, the standard deviation of the time bin is 2 ps with an average value of 203.8 ps. The span of the time bin decreases significantly as shown in the upper side of Figure 14. Note that the global compensation loop (Figure 2) minimizes the deviation of $f_{o}$, and hence $T_{bin}$, due to chip-to-chip process parameters’ deviation. This compensation scheme cannot be applied to cancel the deviations of the TDCs time bin due to pixel-to-pixel mismatches.

The 64 × 64-pixels dToF-CIS has the following specifications:

(i) Pixel size of 64 × 64 μm^2^, fill factor of 2.7%, and in-pixel TDC area of 1740 µm^2^;
(ii) The TDC least significant bit (LSB) variation caused by pixel-to-pixel mismatches is between 146.7 ps and 155.6 ps. It means a standard deviation of 32 codes at full scale. The RMS DNL and INL computed across the array are less than 0.35 LSB and 1.5 LSB [18];
(iii) The single shot precision at 10% and 90% of the full range has a standard deviation of 0.79 and 13.88 codes, respectively;
(iv) The TDC average power consumption of 9 μW has been obtained from post-layout worst-case simulations. In order to have a fair comparison with reference [10], we have normalized it per 10 ns conversion time and 500 k conversions per second;
(v) As has been verified by the experiments, the global compensation scheme considerably reduces the spreading of the TDCs time bin from: (a) 20% down to 2.4% while the temperature ranges from 0 °C to 100 °C; (b) 27% down to 0.27%, when the voltage supply changes within ±10% from the nominal value of 1.8 V; (c) 5.2 ps to 2 ps standard deviation due to process variation, with an average value of 198.2 ps and 203.8 ps. 30 samples have been measured during this experiment.

**Figure 15:**
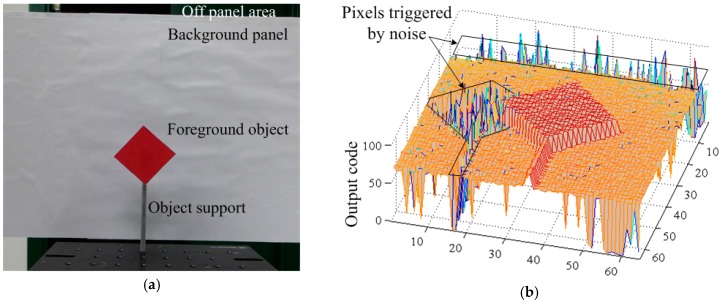
Depth map imaging: (**a**) snapshot of the scene taken by a conventional camera; (**b**) snapshot of the scene taken by the prototype single-photon avalanche-diode (SPAD) camera.

The SPAD camera prototype is aimed at depth map photography. A demonstrative snapshot is shown in Figure 15. The laser illuminates the scene from the right side which explains the shadow on the left side of the picture. Thus no photons from the laser reach back to the sensor. The same occurs to the area from the upper side of the picture (Figure 15b—black marker). The pixels corresponding to these areas are triggered only by uncorrelated noise. 

## 5. Conclusions

Direct ToF-CIS with in-pixel TDCs are sensitive to PVT variations as they lead to deviations of the locally-generated multiphase clock which in turn is inversely proportional to the TDC time bin. Consequently, these variations are causing changes in the gain of the TDC, and hence the output code and distance estimation in 3D ranging applications. This work covers a detailed analysis of these variations and their impact on the performance of the imager. The design and characterization of a global compensation loop against the aforementioned non-idealities is presented as well. The calculations are validated by simulations and/or measurement results. 

## Figures and Tables

**Table 1:** Comparative results.

Parameters	Prediction	Simulation	Measurement NC/C *
$f_{o}$	647.4 MHz	647.4 MHz	630.7 MHz/613.3 MHz
$∆f_{o}$	51.4 MHz	42.28 MHz	16.5 MHz/6 MHz
$T_{bin}$	193 ps	193 ps	198.2 ps/203.8 ps
$∆T_{bin}$	15.3 ps	12.6 ps	5.2 ps/2 ps

* NC = without compensation; C = with compensation

**Table 2:** Model parameters.

Par.	$t_{ox}$ [pm]	$\mu_{n}$ [m^2^/Vs]	$\mu_{p}$ [m^2^/Vs]	$V_{T_{N}}$ [mV]	$V_{T_{P}}$ [mV]	$L_{i}$		$W_{i}$				
						*i = MN, MN1, MN2* [nm]	*i = MP, MP1, MP2* [nm]	*i = MN* [nm]	*i = MN1* [nm]	*i = MN2* [nm]	*i = MP1* [nm]	*i = MP, MP2* [nm]
μ	4200	0.0314	0.0114	307.3	456	169	178	1200	800	250	2399	999.7
σ	28.3	0.000315	0.00011	5.3	6.5	5.7	2.9	6.2	6.2	6.2	5.9	5.9
